# Supplementary figures and images for: Plasma-derived exosome-like vesicles are enriched in lyso-phospholipids and pass the blood-brain barrier
Source: PLoS One. 2020 Sep 21;15(9):e0232442. doi: 10.1371/journal.pone.0232442 (PMC7505448; doi:10.1371/journal.pone.0232442)

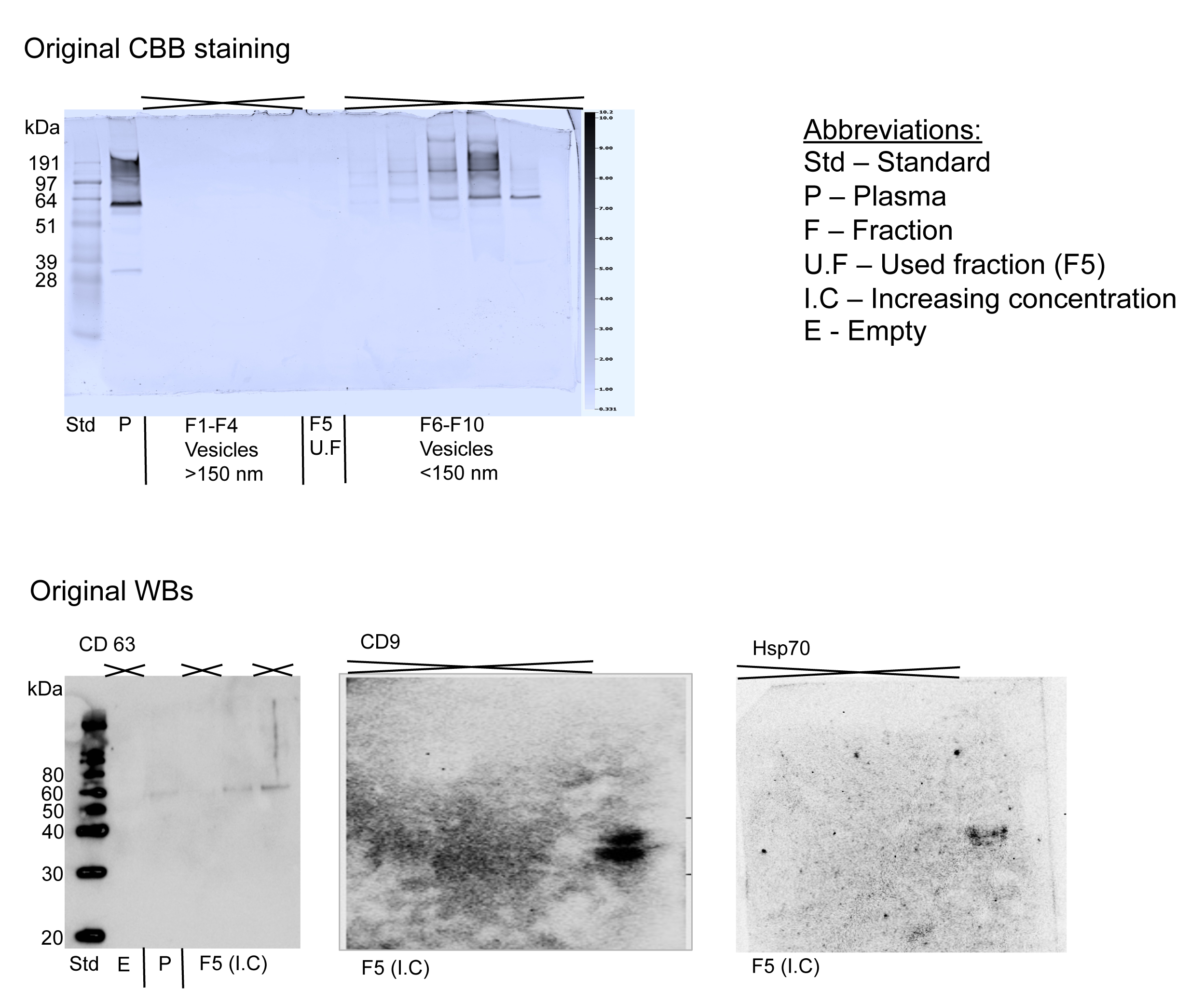

Supplement: S1 Fig — (TIF) [file pone.0232442.s001.tif]

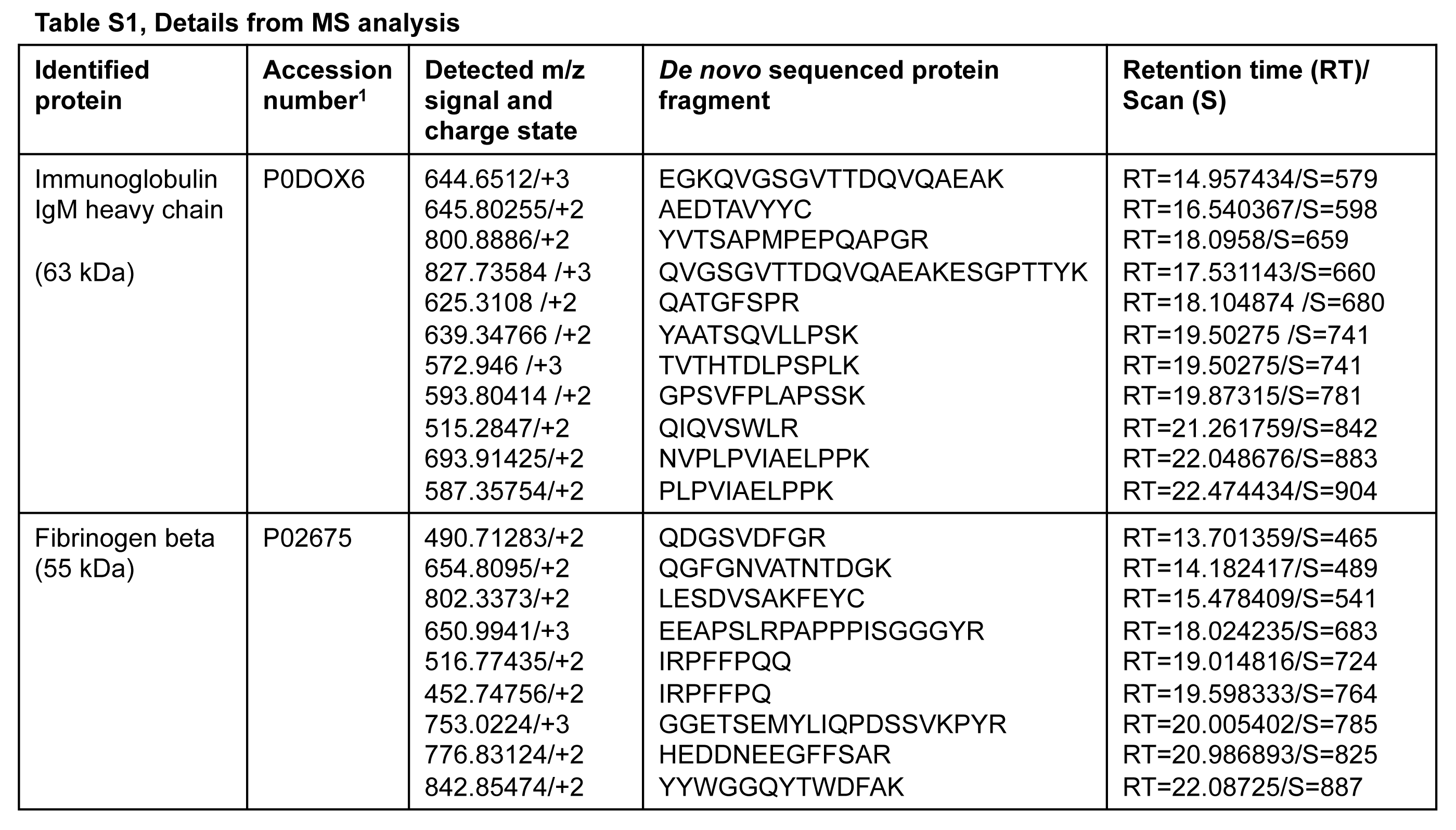

Supplement: S1 Table — (TIF) [file pone.0232442.s002.tif]
