# Supplementary material for: Plasma-derived exosome-like vesicles are enriched in lyso-phospholipids and pass the blood-brain barrier
Source: PLoS One. 2020 Sep 21;15(9):e0232442. doi: 10.1371/journal.pone.0232442 (PMC7505448; doi:10.1371/journal.pone.0232442)
Supplement: Attachment — Submitted filename: Response to reviewers.pdf [file pone.0232442.s004.pdf]

Dear Joe W. Ramos, Ph.D

I here provide our response to the review of:

PONE-D-20-10605
Title - Plasma-derived exosome-like vesicles are enriched in lyso-phospholipids and pass the blood-brain barrier

Journal Requirements:

1. *Please ensure that your manuscript meets PLOS ONE's style requirements, including those for file naming.*

Answer: We have changed the figure file titles, the font size of the major headlines and made sure the whole manuscript is written in Times New Roman. The manuscript now conforms to the journal style.

2. *PLOS ONE now requires that authors provide the original uncropped and unadjusted images underlying all blot or gel results reported in a submission's figures or Supporting Information files. This policy and the journal's other requirements for blot/gel reporting and figure preparation are described in detail at https://journals.plos.org/plosone/s/figures#loc-blot-and-gel-reporting-requirements and https://journals.plos.org/plosone/s/figures#loc-preparing-figures-from-image-files. When you submit your revised manuscript, please ensure that your figures adhere fully to these guidelines and provide the original underlying images for all blot or gel data reported in your submission. See the following link for instructions on providing the original image data: https://journals.plos.org/plosone/s/figures#loc-original-images-for-blots-and-gels.*

Answer: We have attached the original uncropped figure file for the gel and blot in Figs 1A and 1B, and we have made sure that the labeling in the figure adhere to the journal requirements. The file has been renamed and you will now find it attached as "S1_raw_images'.pdf" and sited in the supporting information (Pages 23 and 24, Revised Manuscript with Track Changes)

In your cover letter, please note whether your blot/gel image data are in Supporting Information or posted at a public data repository, provide the repository URL if relevant, and provide specific details as to which raw blot/gel images, if any, are not available. Email us at plosone@plos.org if you have any questions.

Answer: We have now stated in the Cover letter that the blot/gel image data can be found in the supporting information (Pages 23 and 24, Revised Manuscript with Track Changes).

3. *Your ethics statement must appear in the Methods section of your manuscript. If your ethics statement is written in any section besides the Methods, please move it to the Methods section and delete it from any other section. Please also ensure that your ethics statement is included in your manuscript, as the ethics section of your online submission will not be published alongside your manuscript.*

Answer: The ethics statement has been removed from the end of the manuscript (page 18, Revised Manuscript with Track Changes) and does now only appear in the Methods section (page 4, Revised Manuscript with Track Changes).

4. *Please include captions for your Supporting Information files at the end of your manuscript, and update any in-text citations to match accordingly. Please see our Supporting Information guidelines for more information: http://journals.plos.org/plosone/s/supporting-information.*

Answer: We have now inserted captions for our supporting information files at the end of the manuscript (pages 23 and 24, Revised Manuscript with Track Changes).

Reviewer #1

The manuscript by Jakubec M et al., describes the composition and characteristics of extracellular vesicles isolated from the plasma of healthy individuals, and how this affects their ability to cross the blood-brain barrier (BBB). Despite the descriptive nature of this manuscript, there are several points of merit in this study. First, it represents the first extensive characterization of the properties of EVs isolated from the plasma of healthy individuals (which represents the necessary background knowledge to the use of EVs as therapeutics and/or biomarkers); second, it confirms (and quantifies) the ability of circulating EVs to cross the BBB; third, it shows for the first time that plasma EVs loose phosphatidylserine as a membrane component (a finding that is new and could have profound implications in specifically detecting their plasma origin); fourth, it provides a detailed and rigorous analysis of the lipid composition of EV membranes.
Only one minor comment: The Authors should provide the exact catalog number of the antibodies used for their EV characterizations.

Answer: We agree with the reviewer and have now provided the exact catalog number of the antibodies in the Methods part of the manuscript (Page 5, in Revised Manuscript with Track Changes).

Reviewer #2:

Jakubee et. al., describe the isolation of exosomes from the plasma and its lipid components. The manuscript in general is well written and includes all the detail about the methods and approach. Reviewer has few minor comments:

1) The blots included in Figure 1B are not very clear and authors should improve the blots, probably better quality can be achieved by slot/dot blots.

Answer: We agree with the reviewer that the blots should be improved. However the need to collect more plasma samples are very challenging due to the ongoing pandemic. The original blots provided in file S1_raw_images'.pdf, show that although there is background noise, it is still well possible to differentiate signal and noise. We therefore hope that the reviewer can accept the blots as they are.

2) Figure 1 is lacking statistical analysis for every panel. Authors should include this information.

Answer: We partially agree with the reviewers comment. In panel 1A, we have reported on the presence of protein in general by a coomassie brilliant blue stain. We do not use the data in panel 1A to support a quantitative statement and therefore we feel that statistical analysis is not needed. In panel 1B, we have presented the identification of the exosome specific proteins CD63, CD9 and Hsp70. We do not use the data in panel 1B to support any quantitative statement and therefore we feel that statistical analysis is not required. For panel 1C, we agree with the reviewer that some statistical analysis should have been included. We have now included error bars for three independent isolations of EVs. For the DLS analysis in panel 1D, we have presented the mean hydrodynamic diameter of three independent vesicle isolations with three technical replicates. This has now been described in the methods part (Page 5, in Revised Manuscript with Track Changes) for clarity. We have not presented error bars in the figure due to the consecutive nature of the data on the X-axis. We had however, presented the mean hydrodynamic diameter and polydispersity index with standard deviations in the text (Pages 11 and 12, Revised Manuscript with Track Changes).

3) For Figure 3, it is not clear the reviewer if any positive control was used to conclude that exosomes isolated from plasma do not containing PS.

Answer: We agree with the reviewer that it was not clear if we had tested the method on controls containing PS. We have now included the information on the Avanti Lipid MAPS standards used in the relevant methods paragraph (Page 9, Revised Manuscript with Track Changes).

4) *For Figure 2, please include quantization and scale for images.*

Answer: The scale bare was included in the figure legend, but we have now added the value to the line in the image as requested. We have not performed a quantization of the analysis in Fig 2, we have therefore made sure not to state any quantitative claims in the text relating to the data.
